# Variability in Bioreactivity Linked to Changes in Size and Zeta Potential of Diesel Exhaust Particles in Human Immune Cells

**DOI:** 10.1371/journal.pone.0097304

**Published:** 2014-05-13

**Authors:** Srijata Sarkar, Lin Zhang, Prasad Subramaniam, Ki-Bum Lee, Eric Garfunkel, Pamela A. Ohman. Strickland, Gediminas Mainelis, Paul J. Lioy, Teresa D. Tetley, Kian Fan Chung, Junfeng Zhang, Mary Ryan, Alex Porter, Stephan Schwander

**Affiliations:** 1 Rutgers School of Public Health, Department of Environmental and Occupational Health, Piscataway New Jersey, United States of America; 2 Department of Chemistry and Chemical Biology, Rutgers University, Piscataway, New Jersey, United States of America; 3 Rutgers School of Public Health, Department of Biostatistics, Piscataway, New Jersey, United States of America; 4 Department of Environmental Sciences, Rutgers University, New Brunswick, New Jersey, United States of America; 5 Environmental and Occupational Health Sciences Institute Rutgers University, Piscataway, New Jersey, United States of America; 6 National Heart and Lung Institute, Imperial College, London, United Kingdom; 7 Nicholas School of the Environment and Duke Global Health Institute, Duke University, Durham, North Carolina, United States of America; 8 Department of Materials and London Center for Nanotechnology, Imperial College, London, United Kingdom; 9 Rutgers School of Public Health, Center for Global Public Health, Piscataway, New Jersey, United States of America; 10 Robert Wood Johnson Medical School, Rutgers University, Piscataway, New Jersey, United States of America; Duke University Medical Center, United States of America

## Abstract

Acting as fuel combustion catalysts to increase fuel economy, cerium dioxide (ceria, CeO_2_) nanoparticles have been used in Europe as diesel fuel additives (Envirox™). We attempted to examine the effects of particles emitted from a diesel engine burning either diesel (diesel exhaust particles, DEP) or diesel doped with various concentrations of CeO_2_ (DEP-Env) on innate immune responses in THP-1 and primary human peripheral blood mononuclear cells (PBMC). Batches of DEP and DEP-Env were obtained on three separate occasions using identical collection and extraction protocols with the aim of determining the reproducibility of particles generated at different times. However, we observed significant differences in size and surface charge (zeta potential) of the DEP and DEP-Env across the three batches. We also observed that exposure of THP-1 cells and PBMC to identical concentrations of DEP and DEP-Env from the three batches resulted in statistically significant differences in bioreactivity as determined by IL-1β, TNF-α, IL-6, IFN-γ, and IL-12p40 mRNA (by qRT-PCR) and protein expression (by ELISPOT assays). Importantly, bioreactivity was noted in very tight ranges of DEP size (60 to 120 nm) and zeta potential (−37 to −41 mV). Thus, these physical properties of DEP and DEP-Env were found to be the primary determinants of the bioreactivity measured in this study. Our findings also point to the potential risk of over- or under- estimation of expected bioreactivity effects (and by inference of public health risks) from bulk DEP use without taking into account potential batch-to-batch variations in physical (and possibly chemical) properties.

## Introduction

Diesel exhaust particles (DEP) can contribute up to 40% of the total mass of urban ambient aerosolized particulate matter (PM), and therefore, are of major concern for public health as well as environmental monitoring agencies and the automobile industry [1]. Large fractions of the particles comprising the total DEP mass are in an aerodynamic diameter range of <2.5 µm (PM_2.5_) [2] and can reach pulmonary alveolar spaces. Inhalation of PM_2.5_ results in physical interactions between the particles themselves on the one hand and the particles and lining fluid as well as the cellular immune system of the respiratory tract on the other hand. Particularly, the ultrafine components of the PM_2.5_ are sufficiently small (<100 nm) to cross the lining fluid and respiratory epithelial cell layers and, thus, could potentially gain access to the systemic circulation, i.e. the blood stream, resulting in adverse health effects in remote organs [3]–[8].

DEP, a model for primary PM, have been studied extensively for their toxicity, bioreactivity, and health effects in experimental and observational studies [9]. DEP have also been shown to induce toxicity both *in vitro* and *in vivo* in animals as well as in humans [10]–[18]. In addition, DEP are known to alter the allergic disposition and host immune responses to bacteria by inducing a switch from a baseline Th1 to a Th2 cytokine profile [19], [20], and by reducing the capacity of mice co-exposed to DEP and *Mycobacterium tuberculosis* (*M.tb*) [21] or *Listeria monocytogenes* infection [22], [23] to control the bacterial growth in their lungs.

Attempts to reduce the environmental impact of DEP include innovations in engine design technologies, improvements of diesel fuels, and the use of fuel additives [24], [25]. Recently, cerium dioxide (CeO_2_) nanoparticles have been used as a fuel-borne catalyst in Europe and elsewhere [24], [25]. The primary motivation for using nanotechnology-based diesel additives like CeO_2_ is to increase the engine combustion efficiency and thereby saving fuel (www.energenics.co/uk/category/case-studies/). However, it remains to be determined whether the addition of CeO_2_ nanoparticles, which can lead to changes in physicochemical properties of the DEP [26], alters the toxicity and bioreactivity of DEP [9]. The original goal of the current study was to compare the effects of particles (DEP) from a diesel engine (electrical power generator) combusting a regular diesel fuel with particles (DEP-Env) from the same engine combusting the diesel fuel with a commercial CeO_2_ diesel additive (Envirox, www.energenics.co/uk/category/case-studies/). Furthermore, since one effect of CeO_2_ diesel additive Envirox to the diesel fuel is the alteration of particle size, we hypothesized that CeO_2_ addition would have an impact on bioreactivity. Immunocytotoxicity and bioreactivity of DEP and DEP-Env were assessed in THP-1 cells, a human monocytic leukemia cell line and primary human peripheral blood mononuclear cells (PBMC). Furthermore, to assess effects of DEP and DEP-Env on pathogen-specific functional immune responses, cells were co-exposed to DEP or DEP-Env and the respiratory pathogen *M.tb*. During the course of the study, we used DEP and DEP-Env from three independent batches that were collected, stored, and extracted at different times following the same protocols. Surprisingly, we observed large batch-to-batch variations in physical properties (size and surface charge) of as-exposed DEP and DEP-Env for the same fuel-Envirox combinations as well as in the DEP and DEP-Env-induced bioreactivity and functional immune response profiles. These batch-to-batch variations hampered our ability to address our original goal of assessing the effect of the presence of CeO_2_/Envirox at different concentrations in diesel fuel. However, an important and crucial discovery was that across the three collections (batches), a marked correlation between physical properties of DEP and DEP-Env (i.e. size and zeta potential) and bioreactivity (induction of an immune response) was observed.

These novel observations indicate that variability in the physical characteristics of DEP, which may have been collected and processed using different protocols across different studies, are likely to produce inconsistent findings. The current work has important implications in particular for experimental studies in biological systems that often examine aged bulk DEP without prior knowledge of its physicochemical properties.

## Methods

### Collection of Diesel Exhaust Particles (DEP)

All DEP generation and batch collections were conducted in the Controlled Environmental Facility (CEF) at the Environmental and Occupational Health Sciences Institute (EOHSI) of Rutgers University. The diesel exhaust generation system is an integrated part of the CEF. The diesel exhaust generation system consists of a diesel generator (Model YDG 5500E, Yanmar Inc.) and a stainless steel piping delivery and dilution system. The generator was operated at 100% load using heaters. The system used a series of control valves to adjust the amount of diesel exhaust between the exhaust stack and the CEF to maintain the desired concentration of particles. The DEP was collected after the exhaust was diluted with filtered CEF intake air at room temperature.

DEP from a regular diesel fuel and those (DEP-Env) from the fuel doped with Envirox (a diesel additive containing CeO_2_ nanoparticles) were generated and collected at three different time points in 2012 (batch #1, #2 and #3). Envirox was added to the regular diesel fuel at concentrations of 0.1, 1, and 10-fold the manufacturer-recommended doping concentrations (hereafter denoted DEP-0.1x, DEP-1.0x, DEP-10x), corresponding to CeO_2_ concentrations in the fuel of 0.9 µg/ml, 9.0 µg/ml, and 90 µg/ml, respectively. The diesel fuel was number 2 diesel (ultra-low sulfur diesel) that was available at any gas station in New Jersey during the DEP collection period in 2012. More detailed characteristics of the fuel, Envirox, the engine, and the DEP generation system can be found in another publication [26].

The exhaust diesel particles were collected onto 37 mm Teflon filters (Pall Corp.-Life Sciences, Ann Arbor, Michigan 48103, USA) using an air sampling pump at a flow rate of 10L/min for 3 hours. Before and after the sample collection, the filters were placed for at least 24 hours in a climate-controlled weighing room and then weighed using a high-sensitivity microbalance (Mettler Model XP6 with a sensitivity of 1 µg) under static-charge-free conditions. After collection, filters were individually placed in Petri dishes, wrapped with aluminum foil to avoid light exposure, and then placed in a -20°C freezer. Samples were collected on 4 different days between January 16 and February 16, 2012 for Batch 1 series (0X, 0.1X, 1X, and 10X), on March 26, 2012 for Batch 2 series, and on May 22, 2012 for Batch 3 series.

### Preparation of DEP Suspension for *In vitro* Studies

DEP and DEP-Env filters were immersed into 10 mL of HPLC-grade water (Sigma Co.) in a glass vial. To avoid microbial contamination, the glass vials were baked in an oven at 120°C overnight prior to use. The vial was then placed into an ultrasonic water bath (Model FS9, 55W, 43 KHZ, Fisher Scientific Co. USA) and sonicated intermittently over a period of 5 days for a cumulative duration of 48 hours. At the completion of filter extraction, filters were taken out of the vial, and the vials containing DEP or DEP-Env water suspensions were stored in a 4°C refrigerator until further use. After extraction filters were left to dry at room temperature before being weighed in the same weighing room. The difference between filter mass before and after the extraction was used to determine the concentration of the DEP or DEP-Env suspensions. The extraction efficiency (the fraction of particles extracted off the filter in the total particle mass collected on the filter) ranged from 91 to 95%. Prior to use in cell exposure studies, the vials containing DEP or DEP-Env suspensions were sonicated for 5 minutes to ensure uniform dispersion, then aliquots were taken and added to the culture medium at desired concentrations (doses).

### Characterization of DEPs for Size and Surface Charge

The hydrodynamic size and zeta potential of DEP were measured using the Zetasizer (ZS Nano) dynamic light scattering instrument (Malvern Instruments, Malvern, UK) at 25°C with a detection angle of 90°. DEP and DEP-Env samples were sonicated for 5 minutes using a bath sonicator (Branson Electronics) and then diluted to a final concentration of 50 µg/mL in phosphate-buffered saline buffer (pH = 7.4). The number-averaged size and zeta potential values of the samples were then measured using the Zetasizer and averaged over 11 runs (replicates) for size measurements and 13 runs for the zeta potential measurements.

### Endotoxin Analysis

DEP and DEP-Env samples from batch #1-3 were tested for endotoxin contamination by kinetic turbidity Limulus Amoebocyte Lysate [LAL reagent, Associates of Cape Cod (ACC, East Falmouth, MA), T0051)] assay. DEP and DEP-Env samples were diluted with LAL reagent water (ACC, WP0501) to a final concentration of 1.0 mg/mL and the endotoxin concentration was analyzed on the PyrosFlex instrument. *E.coli* lipopolysaccharide supplied by ACC was reconstituted in LAL reagent water and used for the generation of standard curve. Assay internal controls and standards were run with each analysis and were within the USP and FDA requirements of LAL tests [27].

### Study Subjects

Approval to perform this study, collect personal health information, and perform venipunctures was given by the Institutional Review Boards of the University of Medicine and Dentistry of New Jersey (UMDNJ) in Newark and New Brunswick (IRB protocol number 0220100112). After obtaining the written informed consent, peripheral venous blood was obtained by venipuncture from three healthy donors (two female and one male, mean age 45.6 [min. 26, max. 56] years).

### Cell Cultures

#### Preparation of THP-1

THP-1 cells (Cat. No. TIB-202) were obtained from American Type Culture Collection (ATCC) and cultured in RPMI1640 supplemented with Penicillin/Streptomycin/Glutamine and 10% fetal bovine serum (Hyclone Laboratories, Inc. Logan, Utah) and 0.05 mM 2-mercaptoethanol. Cells were maintained at 37°C in humidified 5% CO_2_ environment. Cells were passaged every 3–4 days. Cells were maintained for 4–6 weeks in culture and then discarded.

#### Preparation of Peripheral Blood Mononuclear Cells (PBMC)

PBMC were prepared from whole heparinized venous blood by Ficoll gradient centrifugation [28]. Whole blood was diluted 1∶1 with L-glutamine-supplemented RPMI 1640 medium and centrifuged over a Ficoll-Paque density gradient (1200 rpm, 45 min, and 21°C). Following removal from the interface with Pasteur pipettes, PBMC were washed two times in RPMI 1640, and then resuspended in culture medium (RPMI1640 + L-glutamine + 10% pooled human AB serum). PBMC were then counted, and adjusted to required concentrations. The viability of PBMC was consistently between 98–100% by trypan blue exclusion.

### Preparation of DEP and DEP-Env for *in vitro* Exposure Studies

To assess the effects of DEP and DEP-Env on cell viability and bioreactivity, DEP and DEP-Env (DEP-0.1x, DEP-1.0x, DEP-10x) samples from three independent collections were diluted in complete culture media (RPMI1640 supplemented with Penicillin/Streptomycin/L-Glutamine and 10% pooled human AB serum) following sonication in Branson 3510 water bath sonicator for 2 minutes.

### Viability Assays

Cell proliferation, as a marker of viability, was assessed by Cell Titer 96 Aqueous One Solution Cell Proliferation Assay [MTS, (3-(4, 5-dimethylthiazol-2-yl)-5-(3-carboxymethoxyphenyl)-2-(4-sulfophenyl)-2H-tetrazolium) catalogue number G3580, Promega, Madison, WI]. PBMC (100,000/well) were incubated with 0, 1, 10 and 50 µg/mL of DEP and DEP-Env in 96-well tissue culture plates in a total volume of 200 µl per well at 37°C in a humidified CO_2_ environment for 4 and 24 hours. Equivalent amounts of HPLC water (used for preparing DEP and DEP-Env suspensions) were used to dilute DEP and DEP-0.1x, DEP-1.0x, DEP-10x particles to account for any effect water may have on cell viability. Following the incubation period, cells were centrifuged at 250×*g* for 4 minutes and culture supernatants removed. Cell Titer 96 Aqueous One Solution was diluted 1∶6 with phenol red-free RPMI1640 + 5% PHS + P/S/G and added to the cells in 96-well culture plates. After a 1-hour incubation at 37°C in a humidified CO_2_ environment (incubator), optical density (OD) was recorded at 493 nm. The percentage of viable cells was calculated as the ratio of the OD of stimulated PBMC (after the subtraction of background) to the OD of unexposed PBMC (after the subtraction of background) × 100.

In addition, effects of DEP and DEP-Env (DEP-0.1x, DEP-1.0x, DEP-10x) on the viability of PBMC were measured by trypan blue exclusion assay as follows. PBMC (1×10^6^/well) were plated out in 24-well cell culture dishes and incubated with 0, 10 and 50 µg/mL of DEP and DEP-Env samples, respectively, in a total volume of 1 mL per well for 4 and 24 hours at 37°C in a humidified CO_2_ environment. Cells were diluted 1∶1 with trypan blue dye (Life technologies, Grand Island, NY, Cat. No. 15250061) and counted after 4 and 24 hours incubation with DEP and DEP-Env samples with a hemocytometer at a magnification of 400× under a bright field microscope. Numbers of viable and dead (blue) cells were recorded. The percentage of cell death was recorded as the ratio of number of dead cells (blue) to the total number of cells × 100.

### Quantitation of Cytokine Responses

Frequencies of IFN-γ, TNF-α, IL-1β-producing cells were enumerated with ELISPOT assays as described before [18]. Briefly, 200,000, 100,000 and 20,000 PBMC were incubated with 1 and 10 µg/mL of DEP and DEP-Env samples for IFN-γ, IL-1β and TNF-α, respectively, for 24 hours as recommended by the manufacturer. Following washing with PBS and PBS/0.5% Tween20, appropriate detection antibodies were added to the plates and incubated for 1 hour at 37°C in a humidified CO_2_ environment for IL-1β and overnight at 4°C for IFN-γ and TNF-α. Following washing with PBS/0.5% Tween20, spots were visualized with peroxide–conjugated streptavidin, HRP and chromogen 1% 3-amino-carbamizole. After washing, plates were developed and frequencies of cytokine-producing cells were analyzed by computerized image analysis using a Series 5 Macro reader with software 6.4.82 (C.T.L, Cleveland, OH) as described previously [18].

### Quantitative RT-PCR to determine Cytokine mRNA Expression

To assess the abundance of mRNAs encoding *TNFa*, *IL1b*, *IL6*, and *IL12p40*, PBMC (2×10^6^) were incubated with 1 and 10 µg/mL of DEP or DEP-Env samples for 4 hours at 37°C in a humidified CO_2_ environment. TNF-α, IL-1β, IL-6, and IL-12p40 are proinflammatory cytokines with important functions in protective antimycobacterial human host immunity. Total RNA was extracted from stimulated cells with RNeasy mini columns (Qiagen) as described [18]. Briefly, 250 ng of total RNA was transcribed into cDNA with Taqman reverse transcription reagents (applied Biosystems). cDNA was analyzed by qRT-PCR to determine the abundance of mRNAs encoding *TNFa*, *IL1b, IL6*, and *IL12p40*. The following primer sets were used for qRT-PCR, *TNFa* forward: GTGCTTGTTCCTCAGCCTCTT, *TNFa* reverse: ATGGGCTACAGGCTTGTCATC; *IL1b* forward: GAAGCTGATGGCCCTAAACAG, *IL1b* reverse: AGCATCTTCCTCAGCTTGTCC; *IL6* forward: AGACAGCCACTCACCTCTTCA, *IL6* reverse: CACCAGGCAAGTCTCCTCATT; *IL12p40* forward: GGTGGCTGACGACAATCAGTA, *IL12p40* reverse: TCCTTGTTGTCCCCTCTGACT.

### Bioreactivity Assessments

For *in vitro* cell exposure (bioreactivity studies), DEP, DEP-0.1x, DEP-1.0x, and DEP-10x were suspended into cell culture media by sonication of a stock suspension as described above (2 minutes in water bath sonicator). *In vitro* cell exposure experiments (viability and bioreactivity studies) were performed with DEP, DEP-0.1x, DEP-1.0x, and DEP-10x at concentrations ranging from 0 to 50 µg/mL.

### *M.tb* Exposure

PBMC were infected with avirulent *M.tb* (ATCC strain H37Ra, Cat. No. 25177) at a multiplicity of infection (MOI) of 10. MOI is the ratio of *M.tb* bacteria to blood monocytes within the PBMC.

### Statistical Methods

Mixed linear models were used to assess the association between either particle size or zeta potential and cytokine mRNA expression (*IL6*, *TNFa*, *IL12p40* and *IL1b*). A random effect for donor ID was included in order to account for potential correlation between observations from the same donor. Because there were a few particle sizes that were virtually identical, particle sizes were grouped into bins of 10 nanometers (50–59.9, 60–69.9, etc.). Values for zeta potential were distinct and not collapsed. Particle size and zeta potential were treated as categorical variables within the linear model. An F-test was used to determine whether particle size or zeta potential had any association with the log-transformed cytokine values, followed by all pairwise comparisons between particle sizes or zeta potentials. Log-transformations were applied to the outcomes in order to stabilize the variance and normalize the distribution of cytokine expression. Separate analyses were conducted for mRNA expressions in response to 1 and 10 µg/mL exposures. In order to assess whether the additive effect was the same across collections, we used mixed linear models that included main effects for batch ID, sample ID as well as the cross-product. Sample ID was given values of 1, 2, 3 and 4 and included as a continuous variable. Specifically, an F-test of the cross-product was used to assess the hypothesis of interest. Again, random effects for donor ID accounted for correlations between cytokine levels from the same donors. The effect of each dose of additive (DEP-Env) was assessed individually and in aggregate within each collection. For these analyses, mixed linear models included presence of each level of additive (DEP-Env) as a categorical variable (rather than continuous), using contrasts to compare the cytokine responses following exposure to DEP-Env at each level of additive to cytokine responses following exposure with no additive.

## Results and Discussion

### Physical Properties of DEP and DEP-Env

Hydrodynamic sizes and zeta potentials of DEP and DEP-Env are shown in Tables 1 and 2, respectively. DEP-0.1x appeared to have the smallest mean diameters in all the three batches. The mean particle diameters for batch #3 increased with increasing Envirox concentrations and were markedly different from those of batches #1 and #2. Within the same fuel-Envirox combination (0.1x, 1x, and 10x), large batch-to-batch variations were found, especially between batch #3 and batches #1 and #2. Batch-to-batch variations were also observed in zeta-potential.

**Table 1:** Size of DEP and DEP-Env from Batches # 1-3.

Batch #	DEP-0	DEP-0.1x	DEP-1x	DEP-10x
1	143.2±17.9	57.7±8.7*	74.9±13.5*	97.9±15.1
2	104.5±11.9	74.4±9.0	85.7±10.0	99.5±6.2
3	123.2±14.3	120±14.8	153.3±19.7	278.4±56**

The hydrodynamic size DEP and DEP-Env from batch # 1, 2 and 3 was measured as described in METHODS. Mean particle diameter (nm) ± standard errors from 11 replicates for each sample are listed above. Batch #1 * *p*<0.003 relative to DEP-0. Batch #3 ** *p*<0.01 relative to DEP-0 (2-tailed unpaired t-test with 95% confidence level).

**Table 2:** Zeta potential (mV) of DEP and DEP-Env from Batches # 1-3.

Batch #	DEP-0	DEP-0.1x	DEP-1x	DEP-10x
1	−35.9±2.1	−36.7±2.0	−39.4±2.1	−37.4±1.8
2	−41±1.9	−38.4±2.1	−37.5±2.3	−31.3±2.0
3	−44±2.3	−48±2.5	−41±2.5	−27.5±2.0

The zeta potential of DEP and DEP-Env from batch # 1, 2 and 3 was measured as described in METHODS. Mean particle zeta potential (mV) ± standard errors from 12 replicates for each sample are listed above.

Batch #2 * *p*<0.002 relative to DEP-0. Batch #3 ** *p*<0.0001 relative to DEP-0 (2-tailed unpaired t-test with 95% confidence level).

Although identical collection and extraction protocols were followed, the three batches of DEP and DEP-Env were collected in different months and extracted after different storage durations on the filters. Variations in engine combustion on different collection dates may have contributed to variations in physicochemical properties of as-collected DEP and DEP-Env. In addition, a longer duration of filter storage may have increased agglomeration of particles and resulted in loss of a larger amount of the more volatile components of DEP or DEP-Env. We set out to assess the origins of these variations in DEP properties using the existing data but our evaluations were inconclusive. Exploring the reasons for combustion variability was beyond the scope of this work but are a topic for further investigation.

### Effect of DEP and DEP-Env Exposure on Viability of PBMC

**Figure 1:**
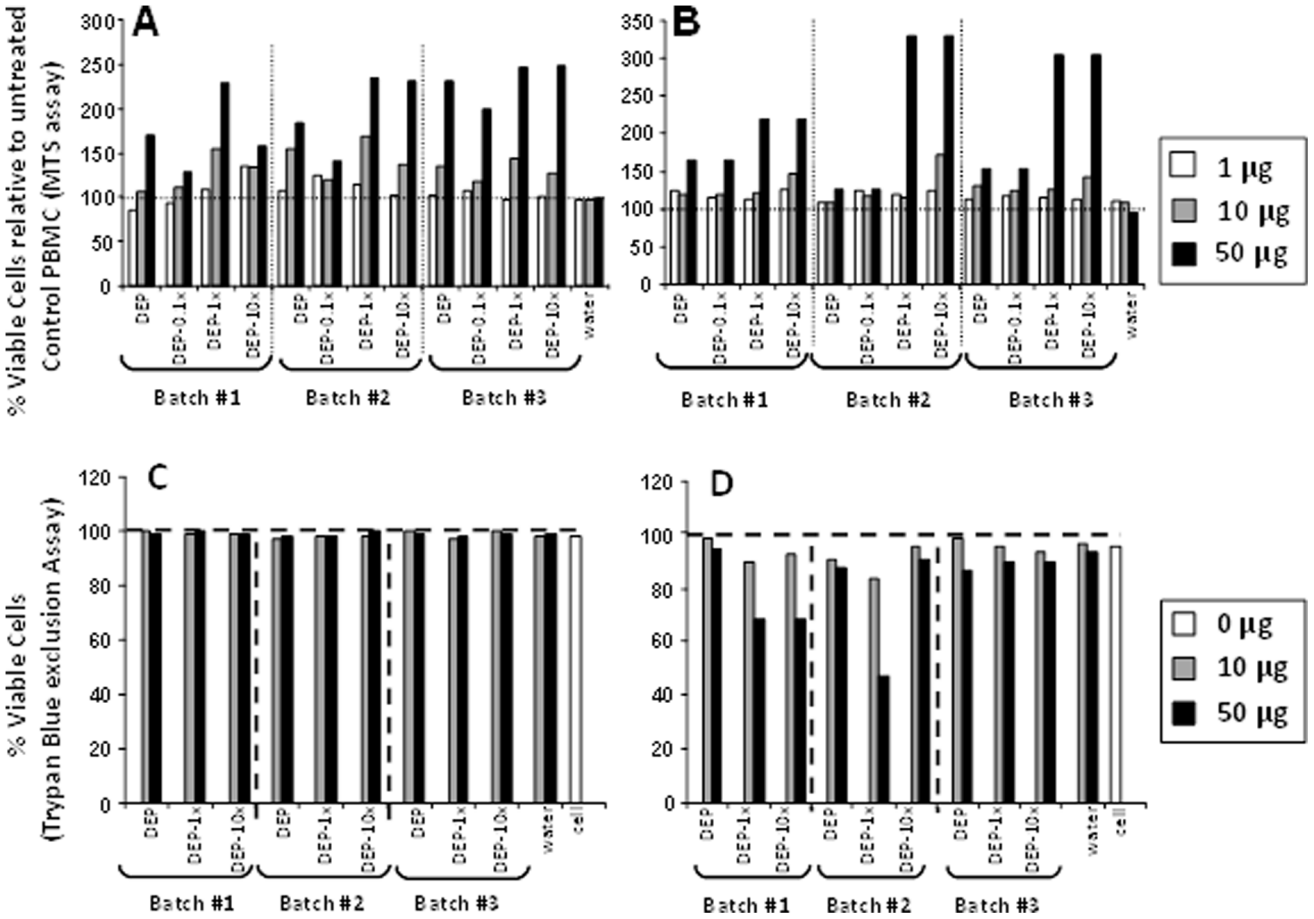
Effect of DEP and DEP-Env Exposure on the Viability of PBMC. Human PBMC (100, 000/well) from a healthy donor were exposed to indicated amounts of DEP, DEP-0.1x, DEP-1.0x, and DEP-10x from three independent batches for 4 (**A**) and 24 hours (**B**). Metabolic activity of cells (proportional to the number of viable cells) was evaluated by MTS assay (**A, B**). PBMC exposed to no particles served as controls. Percent (%) viability of PBMC exposed to DEP and DEP-ENV samples was expressed as the ratio of OD of samples to the OD of unexposed control PBMC x 100. Dashed horizontal lines indicate viability of unexposed PBMC (0 µg, control). Human PBMC (10^6^/well) from a healthy donor were exposed to indicated amounts of DEP, DEP-0.1x, DEP-1.0x, and DEP-10x from three batches for 4 (**C**) and 24 hours (**D**). Numbers of live PBMC were counted with trypan blue and the percentage of viable trypan blue-excluding cells expressed as the ratio of live cells and the total number of cells (live and dead blue cells) x 100. Dashed horizontal lines indicate viability of unexposed PBMC (0 µg, control).

Cellular toxicity in response to exposure to DEP and DEP-Env from batches # 1-3 was evaluated in human PBMC by MTS assay. PBMC were exposed to DEP, DEP-0.1x, DEP-1.0x, and DEP-10x at concentrations of 0, 1, 10 and 50 µg/mL for 4 (Figure 1A) and 24 hours (Figure 1B). These time points reflect the cell culture exposure durations for the assessments of cytokine mRNA and protein expression, respectively. An increase of metabolic activity/viability relative to the unexposed PBMC was observed in PBMC exposed to 50 µg/mL DEP and DEP-Env samples for 4 hours (Figure 1A). After 24 hours, PBMC exposed to 50 µg/mL DEP-Env-1x and DEP-Env-10x from batches #1-3 showed the highest increase in metabolic activity (Figure 1B) as seen at the 4-hour time point. Thus, none of the DEP and DEP-Env samples had any significant cytotoxic effect on PBMC at 4 and 24 hours. However, we cannot ascertain whether increased metabolic activity in PBMC exposed to ≥ 10 µg/mL of particles relative to unexposed PBMC reflects a stimulation-induced increase in cell numbers. In addition, we cannot rule out the possibility that DEP and DEP-Env themselves contributed to increased OD measurements (a measure of increased metabolic activity) at the highest (50 µg/mL) concentration. To validate the results of MTS assays, we assessed the cytotoxicity of DEP and DEP-Env samples on PBMC with trypan blue exclusion assay (Figure 1C and 1D).

PBMC were exposed to DEP, DEP-1.0x, and DEP-10x from batch #1-3 at concentrations of 0, 10 and 50 µg/mL for 4 (Figure 1C) and 24 hours (Figure 1D). The percentage of viable cells was calculated as the ratio of live cells to the total number of cells (live and blue dead cells) × 100. No decrease of viable cell numbers was observed in PBMC exposed to DEP and DEP-Env samples from batch #1-3 for 4 hours relative to unexposed PBMC. However, a decrease in the number of viable cells (25–50%) was observed in cells exposed to 50 µg/mL of DEP-1x and DEP-10x samples from batch #1 and to DEP-1x from batch #2 for 24 hours (Figure 1D). In addition, a slight decrease (<20%) in viability of PBMC was noted following DEP and DEP-1x (batch#1) as well as DEP and DEP1x (batch#2) exposures in Trypan blue assays at 24 hours. No such decrease of viability was observed in MTS assays. Taken together, these results indicate that exposure to 10 µg/mL of DEP, DEP-0.1x, DEP-1.0x and DEP-10x from all three batches at 4 and 24 hours did not induce significant cell death. Based on these results, we subsequently exposed PBMC to DEP and DEP-Env samples at 1 and 10 µg/mL concentrations to examine cytokine protein and mRNA expression profiles.

### Effect of DEP and DEP-Env on Immune Cell Bioreactivity

**Figure 2:**
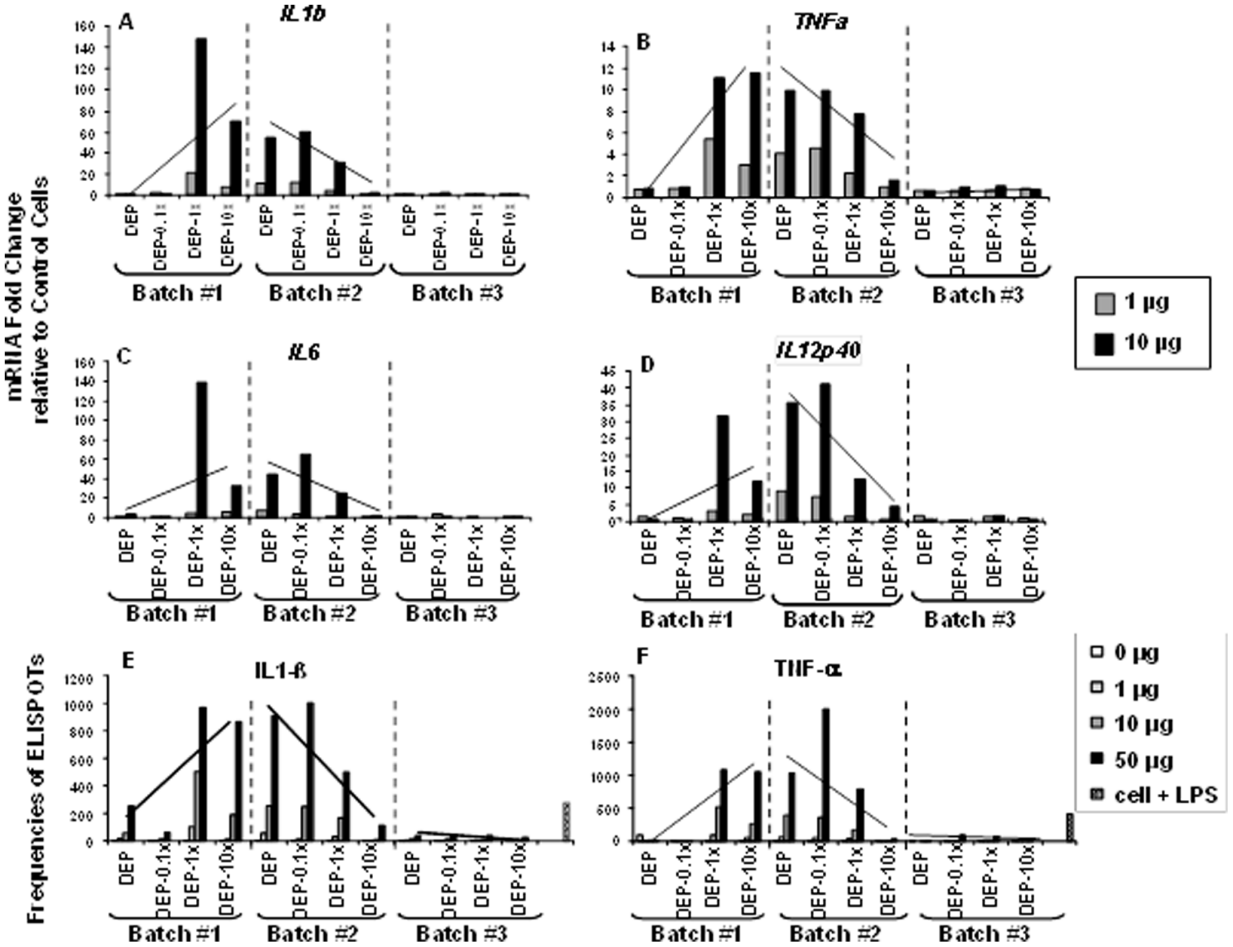
Effects of DEP and DEP-Env Exposure on Cytokine mRNA and Protein Expression in THP-1 Cells. THP-1 cells were exposed to indicated concentrations of DEP, DEP-0.1x, DEP-1.0x, and DEP-10x from three independent batches (#1-3) for 4 hours. The abundance of mRNAs encoding *IL1b*, *TNFa*, *IL6* and *IL12p40* was examined by real-time PCR assays (**A, B, C**, and **D**). Alterations of mRNA levels in THP-1 cells exposed to DEP, DEP-0.1x, DEP-1.0x, and DEP-10x samples are shown as fold changes (y-axes) relative to unexposed (control) THP-1 cells cultured in complete media. THP-1 cells were exposed to indicated concentrations of DEP, DEP-0.1x, DEP-1.0x, and DEP-10x from three independent batches (#1-3) for 24 hours (**E** and **F**). Frequencies of IL-1β and TNF-α cytokine-producing THP-1 cells were assessed by ELISPOT assay. The y-axes represent frequencies of cytokine-producing cells. To demonstrate the change in the expression profile of mRNAs encoding *IL1b*, *TNFa*, *IL6* and *IL12p40* and of IL-1β and TNF-α-producing cells exposed to DEP and DEP-0.1x, DEP-1.0x, DEP-10x from batch #1-3, straight lines indicating the trends are inserted.

***IL1b, TNFa, IL6*** **and** ***IL12p40*** **mRNA Expression.** In a first set of experiments, THP-1 cells were exposed to DEP, DEP-0.1x, DEP-1.0x, and DEP-10x at final concentrations of 0, 1 and 10 µg/mL, and the levels of mRNA encoding *IL1b, TNFa, IL6* and *IL12p40* were compared by qRT-PCR (Figure 2 , panels A, B, C and D). Exposure of THP-1 cells to DEP, DEP-0.1x, DEP-1.0x, and DEP-10x from batch #1 resulted in an increase in mRNA expression for all cytokines examined as the proportions of Envirox relative to DEP increased in DEP-Env samples (DEP-0.1x, DEP-1.0x, DEP-10x). Exposure of THP-1 cells to corresponding concentrations of DEP, DEP-0.1x, DEP-1.0x, and DEP-10x from batch #2, however, resulted in a decrease in mRNA expression for all cytokines examined as the proportions of Envirox increased in DEP-Env samples. In contrast to batches #1 and #2, exposure of THP-1 cells to DEP, DEP-0.1x, DEP-1.0x, and DEP-10x from batch #3 resulted in barely detectable levels of cytokine mRNA as seen in unexposed cells (Figure 2 , panels A, B, C and D).

#### IL-1β and TNF-α Protein Expression

To validate the effects of DEP and DEP-Env samples on cytokine mRNA expression, frequencies of THP-1 cells producing IL-1β and TNF-α were assessed by ELISPOT assay in response to exposure to DEP, DEP-0.1x, DEP-1.0x, and DEP-10x at 0, 1, 10 and 50 µg/mL final concentration (Figure 2E and 2F). As expected, the IL-1β and TNF-α protein expression profiles (Figure 2E and 2F) in response to exposure to DEP, DEP-0.1x, DEP-1.0x, and DEP-10x from batch #1-3 correlated with the corresponding *IL1b* and *TNFa* mRNA expression profiles (Figure 2A and 2B). Exposure to batch #1 DEP-1.0x and DEP-10x samples, appeared to increase the frequency of IL-1β and TNF-α-producing THP-1 cells (relative to DEP) while exposure to batch #2 samples appeared to decrease the frequencies of cytokine-producing THP-1 cells following DEP-1.0x and DEP-10x exposure (relative to DEP). Exposure of THP-1 cells to DEP-0.1x from either batch #1 or #2 did not appear to be significantly different from that of DEP exposed THP-1 cells. Very few IL-1β (Figure 2E) and TNF-α-producing (Figure 2F) THP-1 cells were observed following exposure to batch #3 samples.

### Endotoxin Levels in DEP and DEP-Env Samples

To rule out the possibility that DEP and DEP-Env-mediated bioreactivity was due to endotoxin contamination, we measured the amount of endotoxin in DEP and DEP-Env samples (Table 3). The relatively low levels of endotoxin in DEP and DEP-Env samples from batches #1-3 did not correlate with the bioreactivity of the corresponding samples. Furthermore, previous studies of the effects of endotoxin in these models indicated that this concentration of endotoxin would have no effect. Therefore, observed differences in the bioreactivity resulting from cell exposure to batch #1-3 was unlikely due to the variation in endotoxin contents in different samples.

**Table 3:** Endotoxin Concentrations in DEP and DEP-Env from Batch # 1-3.

Batch #	DEP-0	DEP-0.1x	DEP-1x	DEP-10x
1	2.58	3.68	11.9	2.88
2	8.96	13.1	9.3	26.7
3	8.4	14.2	9.4	10.1

Endotoxin concentrations listed in endotoxin units (EU) per mL of particle suspension were assessed by Limulus amebocyte lysate (LAL) assay in DEP and DEP-Env samples from batch #1, 2 and 3.

### Effects of DEP and DEP-Env on Pathogen-specific Functional Immune Responses

**Figure 3:**
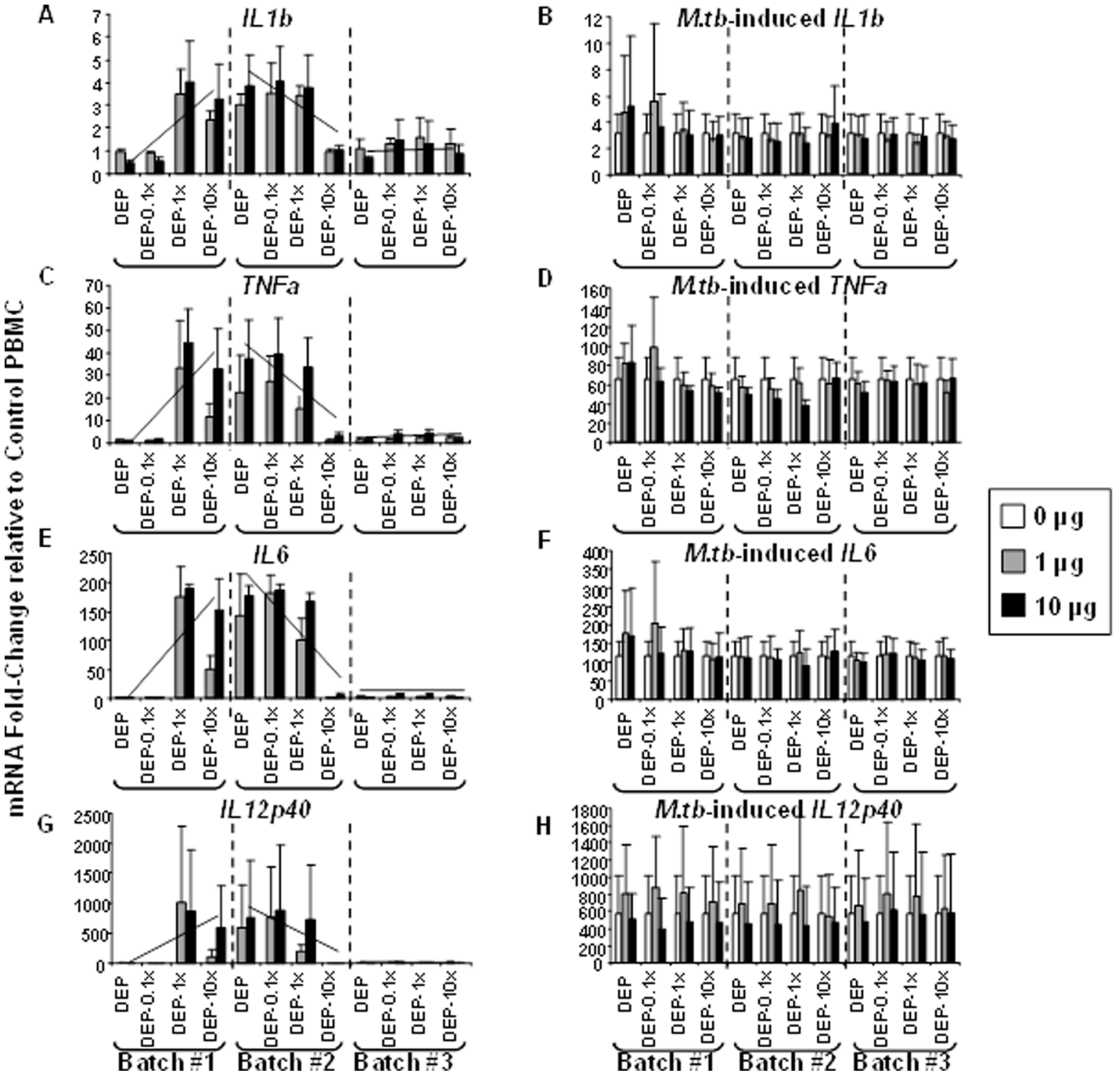
Effects of DEP and DEP-Env Exposure on Cytokine mRNA Expression in Human PBMC. PBMC from healthy donors (n = 3) were exposed to indicated concentrations of DEP, DEP-0.1x, DEP-1.0x, and DEP-10x from three independent batches (#1-3) in absence (**A, C, E**, and **G**) or presence (**B, D, F**, and **H**) of *M.tb* H37Ra at a multiplicity of infection (ratio of *M.tb* bacteria to blood monocytes within the PBMC) of 10 for 4 hours. The abundance of mRNAs encoding *IL1b*, *TNFa*, *IL6* and *IL12p40* was examined by real-time PCR assays as described before. Alterations of mRNA levels in PBMC exposed to DEP and DEP-0.1x, DEP-1.0x, DEP-10x samples are shown as mean fold-changes ± SD (y-axes) relative to unexposed (control) PBMC cultured in complete media. To demonstrate the change in the expression profile of mRNAs encoding *IL1b*, *TNFa*, *IL6* and *IL12p40* in cells exposed to DEP, DEP-0.1x, DEP-1.0x, and DEP-10x from batch #1-3, straight lines indicating the trends are inserted in the left panel.

To assess effects of DEP and DEP-Env on pathogen-specific innate host immune responses, PBMC from three healthy adult blood donors were exposed to DEP, DEP-0.1x, DEP-1.0x, and DEP-10x alone (Figure 3, left panel) or simultaneously to whole viable *M.tb* at a MOI of 10 (Figure 3, right panel). In these experiments *M.tb* served as a public health-relevant pathogen stimulus to examine whether antimicrobial responses of the PBMC would be altered as a result of their simultaneous exposure to *M.tb* and DEP, DEP-0.1x, DEP-1.0x, or DEP-10x. Similar to the findings in THP-1 cells (Figures 2), following exposure to batch #1 samples, mRNA expression of all cytokines appeared to increase in PBMC exposed to DEP-1.0x and DEP-10x relative to DEP-exposed PBMC. In contrast, exposure to batch #2 samples, mRNA expression of all cytokines appeared to decrease in PBMC exposed to DEP-1.0x and DEP-10x relative to DEP-exposed PBMC. PBMC exposed to the batch #3 samples had barely detectable effects on mRNA expression levels for all cytokines relative to unexposed PBMC (Figure 3 , panels A, C, E, and G). Between-batch variability of DEP and DEP-Env samples was apparent in bioreactivity of both the cell line (THP-1) and primary (PBMC) immune cells.

PBMC simultaneously exposed to DEP or DEP-0.1x or DEP-1.0x, or DEP-10x from batch #1-3 and *M.tb* MOI10 (Figure 3 , panels B, D, F, and H) did not follow the *IL1b*, *TNFa, IL6* and *IL12p40* mRNA expression profiles observed for PBMC exposed to DEP and DEP-Env samples alone. The variations in the bioreactivity were less evident in *M.tb*-infected PBMC than in uninfected PBMC. It is worth noting that the co-exposure of PBMC to *M.tb* and DEP or DEP-Env samples led to the expression of much higher levels of *IL1b*, *TNFa, IL6* and *IL12p40* mRNA compared to PBMC exposed to DEP and DEP-Env samples alone. This finding suggests that the strength of the *M.tb*-induced mRNA fold-changes (MOI 10) probably masked the modulatory effects of DEP, DEP-0.1x, DEP-1.0x, and DEP-10x from batch # 1-3 seen without *M.tb* exposure (Figure 3 , panels A, C, E, and G) on the *IL1b*, *TNFa, IL6* and *IL12p40* mRNA expression. Interestingly, when PBMC were stimulated with *M.tb* at MOI1 between-batch differences in cytokine production (IFN-γ, IL-1β, TNF-α) were more clearly visible (data not shown).

**Figure 4:**
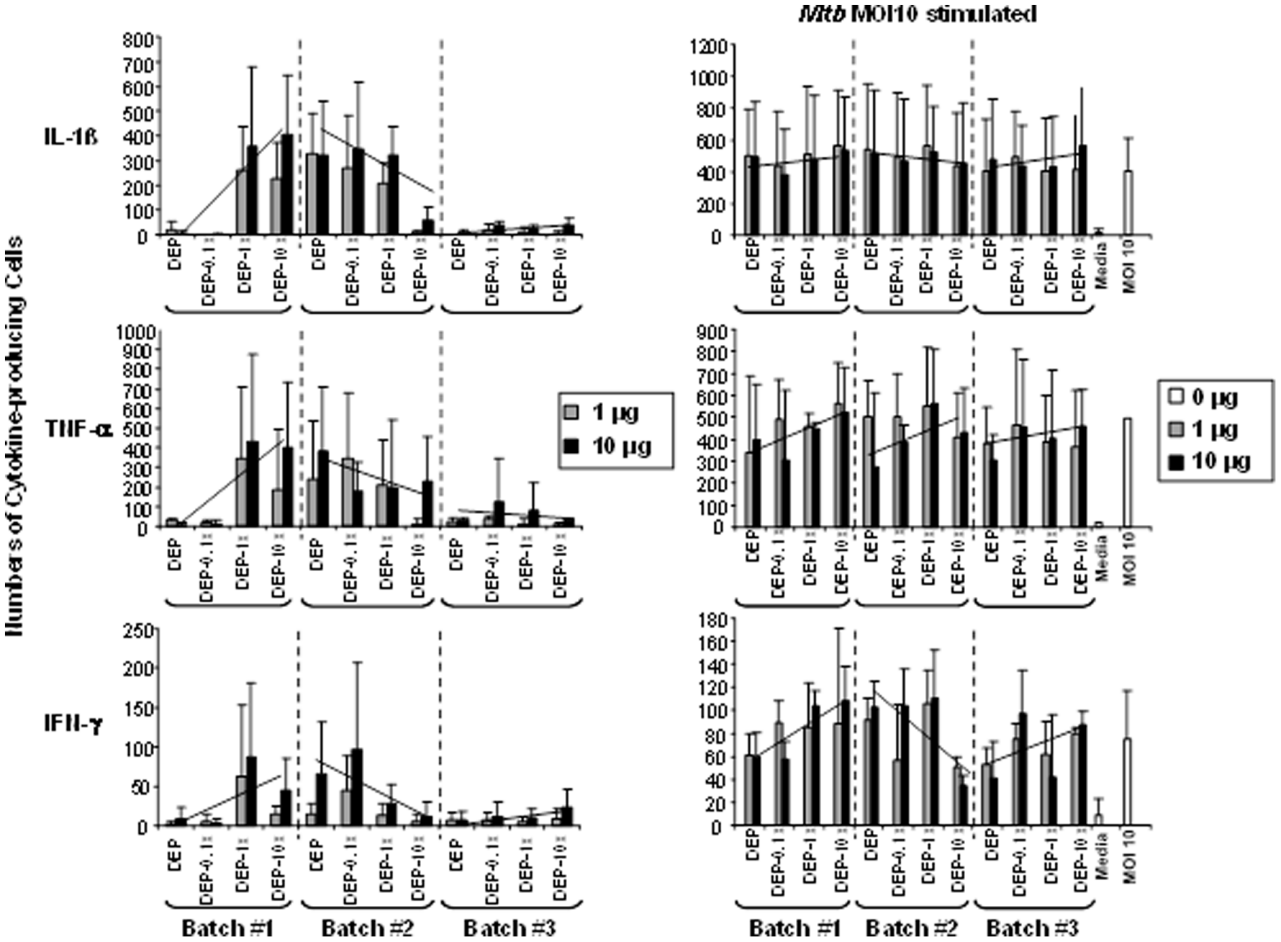
Effects of DEP and DEP-Env Exposure on Cytokine Expression in Human PBMC. PBMC from healthy donors (n = 3) were exposed to indicated concentrations of DEP, DEP-0.1x, DEP-1.0x, and DEP-10x from three independent batches (#1-3) in absence (left panel) or presence (right panel) of *M.tb* H37Ra at a multiplicity of infection of 10 (10 *M.tb* bacteria per blood monocyte within the PBMC) (0 µg/mL) for 24 hours. Frequencies of IL-1β, TNF-α and IFN-γ-producing PBMC were assessed by ELISPOT assay. Y-axes represent cytokine spot frequencies (numbers) of cytokine-producing cells (mean ± SD). To demonstrate the change in the expression profile of cytokine-producing cells exposed to DEP, DEP-0.1x, DEP-1.0x, and DEP-10x from batches #1-3, straight lines indicating the trend are inserted.

In order to examine whether mRNA expression correlates with protein expression, the frequencies of PBMC producing IL-1β, TNF-α and IFN-γ (cytokines important for human antimycobacterial immunity) were determined upon exposure of PBMC to the DEP and DEP-Env samples in the absence or the presence of *M.tb* MOI 10 by ELISPOT assay (Figure 4). Increases and decreases in frequencies of IL-1β, TNF-α-producing PBMC (Figure 4, left panel) followed the pattern that was observed in mRNA levels (Figure 3, left panel ) in response to exposure to DEP, DEP-0.1x, DEP-1.0x, and DEP-10x samples from batch #1-3. While exposure to DEP, DEP-0.1x, DEP-1.0x, and DEP-10x samples from batch #3 resulted in low levels of cytokine protein production, exposure to the batch #1 samples led to an increase in the frequency of IL-1β, TNF-α and IFN-γ-producing PBMC with increasing proportion of Envirox (DEP-1.0x, DEP-10x) relative to DEP-0.1x and DEP. Exposure to the batch #2 samples, however, tended to decrease the frequencies of cytokine-producing PBMC following DEP-0.1x, DEP-1.0x and DEP-10x exposure (relative to DEP) (Figure 4, left panel). Variations in *M.tb*-induced IL-1β and TNF-α mRNA expression (Figure 3, right panel) were not as well-defined as in the protein expression profile (Figure 4, right panel) upon exposure to DEP and DEP-Env from batch #1-3. Upon exposure to the batch #1 and 3 samples, IFN-γ expression appeared to increase in PBMC exposed to DEP-1.0x and DEP-10x relative to DEP-exposed PBMC. In contrast, following exposure to the batch #2 samples, the expression of IFN-γ appeared to decrease in PBMC exposed to DEP-1.0x and DEP-10x relative to DEP-exposed PBMC (Figure 4, right panel).

### Links between Particle Physical Properties and Bioreactivity

**Figure 5:**
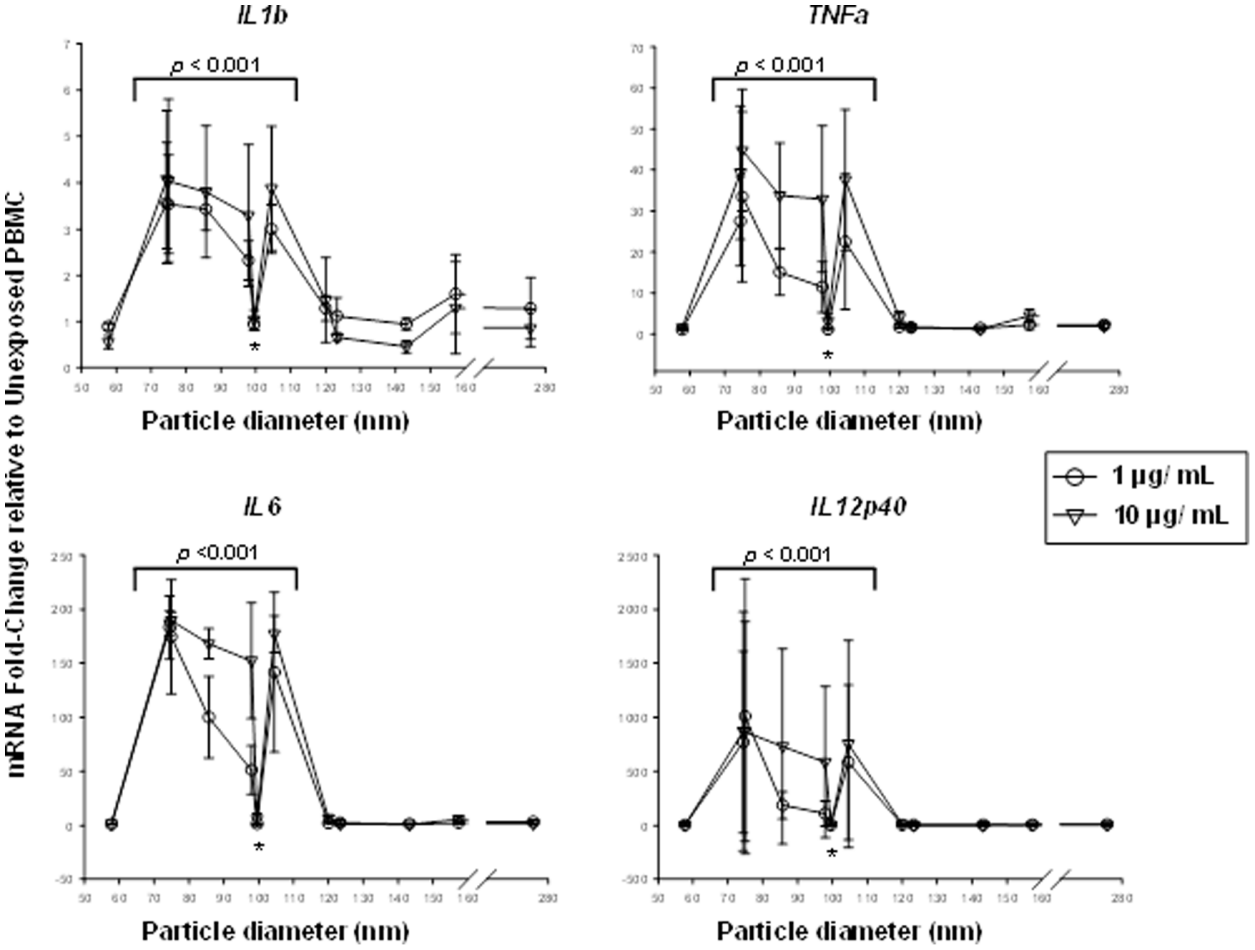
Effect of the Size of DEP and DEP-Env Particles on the Bioreactivity of Human PBMC. *IL1b*, *TNFa*, *IL6*, and *IL12p40* mRNA fold-changes (Y-axes) in PBMC (from 3 donors) shown in Figure 3 are plotted as a function of mean particle diameter in nanometers (x-axes). All particles in the size range from 70 to 110 nm, with one exception (asterisk *), showed significantly higher bioreactivity (*p*<0.001) compared to the particles below or above this size range.

**Figure 6:**
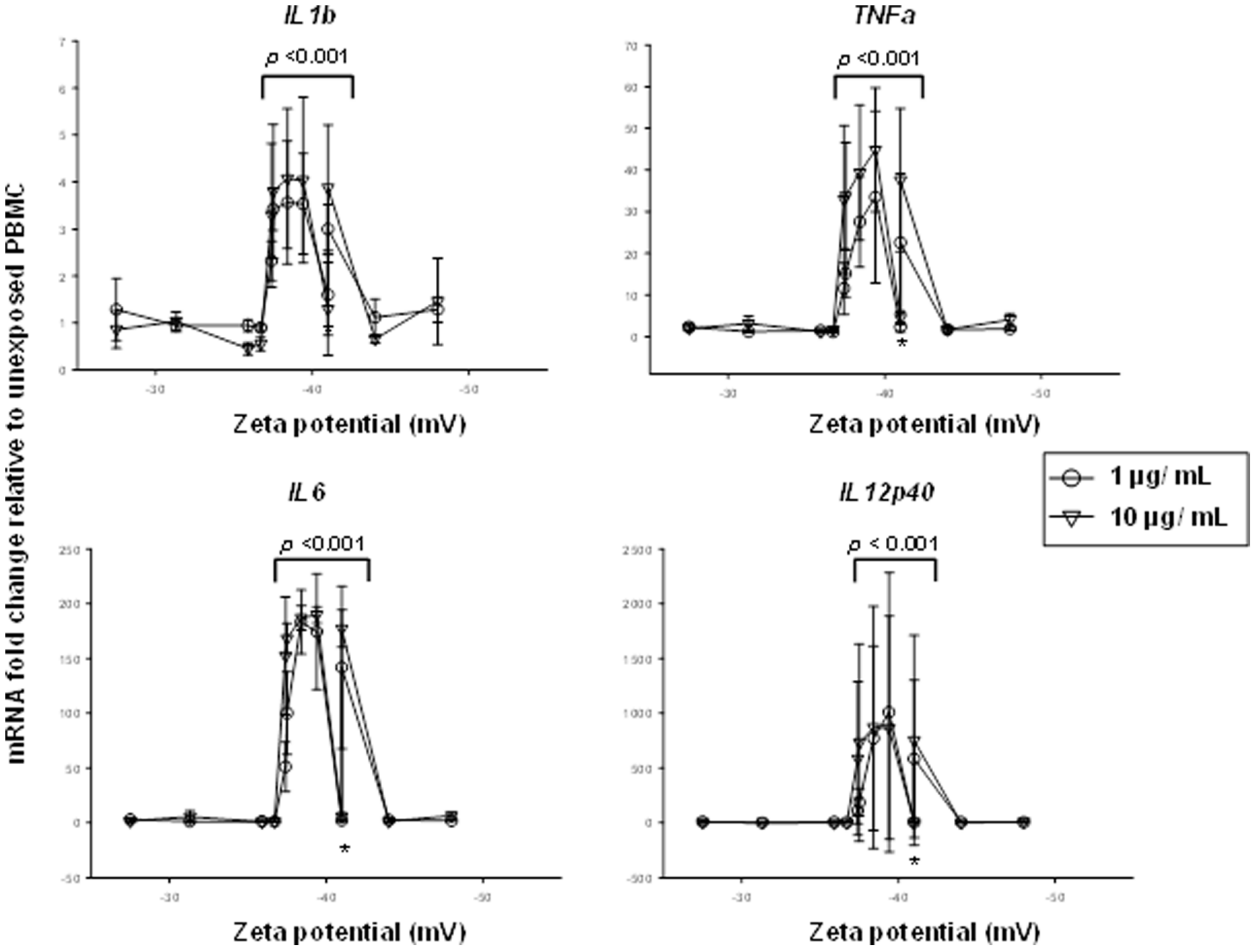
Effect of Charge (Zeta Potential) of DEP and DEP-Env Particles on the Bioreactivity of Human PBMC. *IL1b*, *TNFa*, *IL6*, and *IL12p40* mRNA fold changes (y-axes) in PBMC (from n = 3 donors) shown in Figure 3 are plotted here as a function of mean zeta potential in millivolt (x-axes). All particles with zeta potentials from −37 to −41 mV, with one exception (asterisk *), showed significantly higher bioreactivity (*p*<0.001) compared to the particles below or above this size range.

To examine whether differences in size or zeta potential may have contributed to the observed differences in bioreactivity following PBMC exposures to DEP, DEP-0.1x, DEP-1.0x, and DEP-10x from batch #1-3, we plotted each of the bioreactivity measures against particle size (Figure 5) and zeta potential (Figure 6) using pooled data across all the DEP and DEP-Env samples and across all the batches.

Interestingly, exposure to particles with mean diameters of 60 to 120 nm with one exception resulted in the highest levels of bioreactivity as measured by mRNA fold-changes for *IL1b*, *TNFa, IL6* and *IL12p40* relative to unexposed PBMC (Figure 5). Similarly, high levels of bioreactivity with one exception (different sample from sample that differed in size) were observed in samples with mean particle charges in the range of −45 to −37 mV (Figure 6). Statistical analysis employing mixed linear models to assess associations between either particle size or zeta potential and the expression of cytokine mRNA encoding *IL6*, *IL1b*, *IL12p40* and *TNFa* revealed a significant association of cytokine expression with particle size and zeta potential (see p-values in Figures 5 and 6). Interestingly, for both particle size and zeta potential there is a critical point at which bioreactivity diminishes. It is possible, that there is a particle size range at which the nature of cellular interactions with particles changes, e.g. during particle uptake processes that can change from passive to active. However, we did not observe a clear relationship with endotoxin concentration. Thus, size (diameter) and surface charge (zeta potential) appeared to be the primary determinants of the differences observed in bioreactivity from batches 1–3.

Monocytes in the PBMC are precursors of alveolar macrophages that take up, process and mediate toxicity and immune effects of aerosolized particles in the lung milieu. As-exposed DEP and DEP-Env with the relatively narrow size range (60 to 120 nm) for which we observed bioreactivity, could cross through the respiratory epithelium from the alveolar spaces in the lungs into the systemic circulation, although in low quantities, this could be important during chronic exposure or during significant air pollution episodes [7]. Furthermore, the increased surface area and surface reactivity of DEP in this size range could increase the likelihood of cellular exposure to the soluble surface components of DEP, such as metals and polyaromatic hydrocarbons. This is supported by previous findings that inhalation of diesel exhaust generated *in situ* (containing DEPs in ∼60 to 120 nm size range) has led to extra-pulmonary and systemic effects such as oxidative modifications of high-density lipoproteins (HDLs) in mice [9].

Statistical analysis revealed an association between the size and zeta potential of as-exposed particles and bioreactivity, irrespective of sample batch or DEP and DEP-Env. In all cases, the effects of particle size and zeta potential were highly significantly associated with cytokine expression (all *p*-values<0.0001, Figures 5 and 6). Further, cytokine protein production and mRNA expression levels differed significantly across all batches (all *p*-values <0.0001) for the 1 and 10 µg/mL DEP and DEP-Env exposures. For batches #1 and #2, the slopes of the cytokine protein and mRNA expressions from DEP, DEP-Env-0.1x, 1x and 10x exposures were significantly different from 0 (unexposed cells) for all cytokines. For batch #3, the slope was not significantly different from 0, indicating no effect of the Envirox additions (Figure 3). Further, the slopes following exposure to the particles from batch #1-3 were significantly different from one another (*p*-values for differences overall *p*<.0001). Particle size and zeta potential had a correlation of −0.43 with a *p*-value for a test of no association equal to 0.17. When excluding the outlying particle size (>250), the correlation was only −0.31 with a *p*-value of 0.35. Because the correlation between particle size and zeta potential is relatively weak and the correlations of both particle size and zeta potential with cytokines are strong, both particle size and zeta potential likely have unique effects on the cytokines. We are unable to ascertain the cause for the deviation from the other five data points for the single data points marked by asterisks (*) showing an association between particle size (Figure 5 ) and Zeta potential (Figure 6) and cytokine mRNA expression. However, the outlier (DEP-10x, batch #2, Table 1) in Figure 5 (size) falls outside of the range of Zeta potential where bioreactivity was observed, while the outlier (DEP-1x, batch #3, Table 2) in Figure 6 (Zeta potential) falls outside of the range of sizes where bioreactivity was observed. Thus it appears that particle size and Zeta potential codetermine bioreactivity of the cells.

We are not aware of studies that have linked the impact of particle size and charge changes resulting from DEP collections and extractions at different times with toxicity and bioreactivity in cell exposure or animal models. However, a few studies evaluated the impact of size [29], [30] and charge [30], [31] of PM on bioreactivity in *in vitro* cell model systems. In addition, the source (coal, DEP, oil) and handling of PM were shown to alter the bioreactivity in a rat alveolar epithelial cell line (RLE-6TN) [32].

In the present study, using THP-1 cells and primary human PBMC, we noted significant differences in mRNA expression and protein production profiles among three batches of DEP and DEP-Env samples collected at different times and extracted following different lengths of sample storage. The scope of the present study is limited to evaluating the impact of particle size and charge, due to resource constraints. Potential changes in chemical composition due to differences in collection and extraction times may also contribute to batch-to-batch differences in bioreactivity. Indeed, variation in bioreactivity between DEP samples from different sources depending on their chemical composition has been reported recently [33]. Preparing DEP samples of most real-life exposure relevance for *in vitro* and *in vivo* experimental studies has been and will continue to be a challenging issue [34]–[37].
